# Methodological and statistical approaches for the assessment of foot shape using three-dimensional foot scanning: a scoping review

**DOI:** 10.1186/s13047-023-00617-z

**Published:** 2023-04-27

**Authors:** Jamie J. Allan, Shannon E. Munteanu, Daniel R. Bonanno, Andrew K. Buldt, Simon Choppin, Alice Bullas, Natalie Pearce, Hylton B. Menz

**Affiliations:** 1 Discipline of Podiatry, School of Allied Health, Human Services and Sport, La Trobe University, Melbourne, VIC 3086 Australia; 2 Advanced Wellbeing Research Centre, Sheffield Hallam University, Sheffield, S9 3TU UK; 3 La Trobe Library, La Trobe University, Melbourne, VIC 3086 Australia

## Abstract

**Objective:**

The objectives of this study were to: (i) review and provide a narrative synthesis of three-dimensional (3D) foot surface scanning methodological and statistical analysis protocols, and (ii) develop a set of recommendations for standardising the reporting of 3D foot scanning approaches.

**Methods:**

A systematic search of the SCOPUS, ProQuest, and Web of Science databases were conducted to identify papers reporting 3D foot scanning protocols and analysis techniques. To be included, studies were required to be published in English, have more than ten participants, and involve the use of static 3D surface scans of the foot. Papers were excluded if they reported two-dimensional footprints only, 3D scans that did not include the medial arch, dynamic scans, or derived foot data from a full body scan.

**Results:**

The search yielded 78 relevant studies from 17 different countries. The available evidence showed a large variation in scanning protocols. The subcategories displaying the most variation included scanner specifications (model, type, accuracy, resolution, capture duration), scanning conditions (markers, weightbearing, number of scans), foot measurements and definitions used, and statistical analysis approaches. A 16-item checklist was developed to improve the consistency of reporting of future 3D scanning studies.

**Conclusion:**

3D foot scanning methodological and statistical analysis protocol consistency and reporting has been lacking in the literature to date. Improved reporting of the included subcategories could assist in data pooling and facilitate collaboration between researchers. As a result, larger sample sizes and diversification of population groups could be obtained to vastly improve the quantification of foot shape and inform the development of orthotic and footwear interventions and products.

## Background

Human foot morphology is highly variable and is influenced by a broad array of factors, including age [1], sex [2], ethnicity [3], body mass [4], genetic disorders [5], and musculoskeletal foot conditions such as hallux valgus [6, 7] and osteoarthritis [8]. Foot shape impacts many aspects of an individual's life, including standing balance, movement during walking, sporting performance, predisposition to lower limb injury, and footwear fit [9]. Sub-optimal footwear fit has a significant impact on an individuals comfort, risk of foot pathology development, and falls risk [10].

The impact of variation in foot shape and on footwear fit are significant barriers that consumers, clinicians, and industry face. With the use of three-dimensional (3D) scanning technology, detailed information about the foot can be obtained and analysed to quantify foot shape [11]. This detailed foot shape data can provide researchers with a greater understanding of foot shape across population groups to improve footwear design and fit. Obtaining optimal footwear fit has been increasingly difficult with the continued rise of online purchasing. Currently, there are hundreds of footwear brands on the market, and they do not follow a standardised sizing system [12, 13]. For example, length measurement differences (in the same US size) of 0.5 cm can be observed when comparing a Nike and Adidas running shoe [12]. Difficulties in obtaining correct footwear fit have resulted in higher return rates from online orders due to fit uncertainty, resulting in a negative client experience and increased cost for the company [12, 14]. Additionally, the higher return rates has a significant environmental and economic impact [15].

Although 3D surface scanning has been used to measure foot shape since the mid-1990s [16], only recently has statistical shape modelling using sophisticated morphometric and multivariate statistical analysis techniques been used to identify discrete foot types from 3D shape data [17]. These techniques allow foot shape to be separated from overall object size by using rich 3D data to identify characteristics that are unable to be measured using predetermined 2D measurements [18]. The ability to differentiate foot shape according to sex, age, ethnicity, and pathology has practical applications for footwear design from a structural and functional perspective.

Capturing a 3D model of the foot has been achieved through a variety of methods since its inception. The type of 3D scanning systems have been varied, ranging from the use of stereophotogrammetry (which uses multiple photographs taken from different angles) to structured light (patterns projected on the object) and laser scanning systems (laser/s are repeatedly projected onto a surface while the camera/s and computer system acquires the 3D data) [19]. Early scanning equipment involved using a projector paired with a charged coupled device to capture 3D foot shape [16]. In addition to these methods, smart phone cameras, digitisers, RealSense depth cameras (Microsoft® Kinect; Microsoft, Redmond, WA, USA), and adjustable height pins (Amfit® system, Amfit Inc, Vancouver, WA, USA) have been used to generate 3D foot data [20–22]. Recent studies primarily employ laser scanning technology; however, a wide range of laser scanners are currently available. As a result of the large variety of 3D scanning systems, potential differences in scanner specifications, scanning condition protocols, foot measurements, and statistical analysis techniques may exist between studies. To help overcome some of these issues, 3D surface scanning standards have been created by the International Organisation for Standardisation (ISO) [23] and the Institute of Electrical and Electronics Engineers (IEEE) [24], with the first ISO standard being released in 2015. However, currently it is unclear if these standards have been adopted in the literature.

To the best of our knowledge, with the exception of a broad overview of 3D foot scanning published in 2010 [25], and recent reviews specifically focused on smartphone apps [26] and comparing 3D scanning to traditional methods for fabricating orthoses [27], no studies have consolidated and reviewed the literature pertaining to 3D foot scanning methods in detail. Therefore, the objectives of this paper are to: (i) review and provide a detailed narrative synthesis of 3D foot surface scanning methodological and statistical analysis protocols, and (ii) construct a methodological checklist to help homogenise the reporting of 3D scanning and statistical analysis protocols for future studies.

## Methods

This scoping review was conducted and reported in accordance with the Joanna Briggs Institute methodology for scoping reviews [28] and the PRISMA extension for scoping reviews [29].

### Search strategy

Three electronic databases were searched. An initial limited search of SCOPUS, ProQuest, and Web of Science were undertaken by two independent reviewers (JJA, HBM) to identify key studies for the topic. A reviewer (JJA) and research librarian (NP) identified key text words contained in the titles and abstracts of key studies and these were used to develop a full search strategy for SCOPUS, ProQuest, and Web of Science. Key search terms were grouped into three main concepts and adapted to each database: (i) 3D foot scanning, (ii) shape modelling/analysis/morphology, and (iii) foot/feet. Concept synonyms were searched under ‘topic’ which included title, abstract, and key words. Results from within each concept were combined with ‘OR’ and between concepts were combined with ‘AND’. The search strategy is provided in Supplementary file 1. Manual citation tracking and reference checking of included studies were performed. Grey literature such as conference proceedings were screened for additional studies. Only studies published in the English language were included. Studies published from inception to March 28^th^, 2022, were included. Titles and abstracts were screened by two independent reviewers (JJA and HBM) for assessment against the inclusion/exclusion criteria for the review. Any disagreements on eligibility were resolved at a consensus meeting by a third independent reviewer (SEM).

### Inclusion / exclusion criteria

To be included, papers needed to report studies that employed static 3D surface scanning of the foot. Studies with participants of any age, sex, geographical location, musculoskeletal pathology, or health setting were included. Papers were excluded if the studies used two-dimensional footprints only, 3D scans that did not include the medial arch, dynamic/sequential scans to infer foot movement, derived foot data from a full body scan, or diagnostic imaging techniques that may be used to create 3D models of the foot (such as x-ray, magnetic resonance imaging or computed tomography). Single case studies, studies involving 10 or fewer participants, non-English full text or conference proceedings/abstracts with less than four text pages were excluded due to a lack of detail provided, as were papers which described a scanning technique but provided no data on foot shape, and thesis dissertations. Foot dimensions measured were included if used in two or more studies.

### Study selection and data extraction

Study selection and data extraction were performed independently by two reviewers (JJA and HBM). Any disagreements on study eligibility were discussed between reviewers (JJA and HBM) and resolved at a consensus meeting by a third independent reviewer (SEM). A custom generated data extraction template was created in Covidence (Covidence, Melbourne, Australia). The following individual study data were extracted from included studies: general study information (title, author, database / journal, country, and primary objective), 3D scanner characteristics (name, type, accuracy, and capture duration), study methods (e.g., sample size), 3D scanner methodology (e.g., calibration, data collection, 3D foot measures), scanner reliability (intra- and inter-rater), participant demographics (participant subgroup, age, sex, weight, height, body mass index [BMI], co-morbidities, shoe size, and ethnicity), processing techniques (meshing, smoothing, cropping, scaling, and software used), broad study design, statistical analysis approach, inclusion/exclusion criteria, and main outcomes. All 3D foot measures were cross checked using the IEEE and ISO definitions.

## Results

**Fig. 1:**
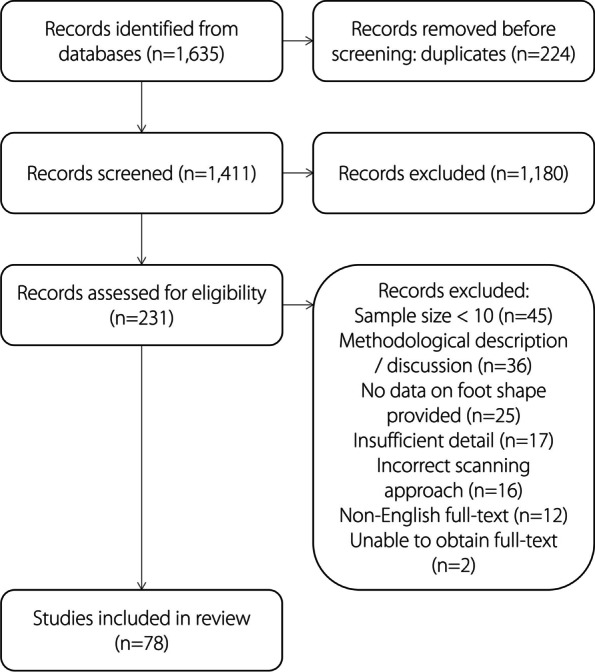
Flowchart of included studies

A flowchart of included studies is shown in Fig. 1. The initial search yielded 1,635 articles; from which 224 duplicates were removed. A further 1,180 articles were excluded in the title and abstract screening with an additional 153 excluded after the full text screening. A final 78 studies were deemed to meet the inclusion criteria.

### Characteristics of included studies

The included studies originated from 17 different countries: China (*n* = 41) [4, 11, 30–68], Japan (*n* = 6) [16, 44, 69–72], Germany (*n* = 4) [13, 73–75], Romania (*n* = 3) [8, 76, 77], South Korea (*n* = 3) [78–80], Spain (*n* = 3) [22, 81, 82], United States (*n* = 3) [83–85], Italy (*n* = 3) [20, 21, 86], India (*n* = 2) [5, 87], Malaysia (*n* = 2) [3, 88], New Zealand (*n* = 2) [35, 62], Australia (*n* = 2) [89, 90], Belgium (*n* = 2) [18, 91], United Kingdom (*n* = 2) [92, 93], Canada (*n* = 1) [94], Iran (*n* = 1) [9], Russia (*n* = 1) [47], Slovenia (*n* = 1) [95], and Sweden (*n* = 1) [75]. Several study designs were utilised in the literature, these included: 51 comparisons of mean differences between groups [3, 5, 8, 9, 22, 30–35, 38–41, 43–45, 47–50, 52–62, 66, 68, 70, 72, 75–78, 80, 83, 85–89, 93–95], 20 cluster/principal component analyses [5, 11, 13, 16, 18, 31, 32, 38, 42, 51, 58, 63, 64, 68, 73, 79, 82, 85, 87, 90], 14 reliability studies [20–22, 34, 37, 39, 48, 49, 53, 75, 78, 88, 90, 91], 10 validation studies [20, 21, 39, 40, 46, 48, 53, 65, 78, 81], one correlation study [33], one comparison of distributions between groups [92], one regression analysis [87], and 11 repeated measures studies were reported, which were grouped into three subcategories; seven studies reported the effect of different loading conditions [4, 22, 36, 39, 74, 84, 90], three reported changes before/after exercise [62, 69, 71], and one reported different alignment methods [67]. Thirty-one studies reported participant inclusion criteria [4, 5, 9, 11, 13, 16, 32, 34, 36, 40, 43, 47, 50, 52, 56, 58, 61, 69, 71, 73, 74, 78, 79, 81, 83, 84, 87, 89, 91, 92, 94] and 30 studies reported exclusion criteria [4, 5, 9, 22, 32–36, 40–42, 46, 47, 52, 61, 67, 69, 71, 73–76, 78, 84, 89–92, 94].

Sample size ranged from 11 to 1,200,847, with 57 studies including healthy young adults [3, 4, 8, 11, 13, 16, 18, 20, 21, 30–40, 42–46, 48–50, 54–56, 59–61, 63–71, 74, 77, 78, 80–84, 87, 88, 90, 91, 93, 94]. Other groups included: older adults (> 65 years) (*n* = 18) [8, 9, 34, 38, 45, 56, 61, 70, 72, 74, 77, 82–84, 87, 91, 93, 94], children/adolescents (< 18 years) (*n* = 11) [5, 13, 37, 38, 41, 52, 56, 75, 80, 89, 92], participants with medical conditions (*n* = 7) (such as Down syndrome [89], diabetes [8, 34, 87], arthritis [8, 94], and cerebral palsy [5]), sports people (*n* = 8) (such as football (soccer) players [73], American football players [85], road cyclists [22], collegiate runners [69, 71], recreational runners [35, 62], and amateur sprinters [50]), non-habitual exercisers [50], pregnant women [4], and habitually barefoot or shod participants [35, 47]. Additionally, five studies included controls in their sample [8, 73, 87, 89, 93], while other studies did not provide specific participant characteristics [51, 58, 79, 86].

All but six studies [21, 53, 57, 65, 73, 79] reported participant sex. Fifty-three studies reported mean age (ranging from 8 to 75 years) [3, 4, 8, 9, 11, 13, 16, 18, 22, 30–36, 38, 40, 43–46, 48, 50, 51, 54–56, 58, 59, 61–64, 67–72, 74, 75, 77, 82–84, 86–90, 93, 94], and 51 reported age standard deviation [3, 4, 8, 9, 11, 13, 16, 18, 22, 30–36, 38, 40, 43–46, 48, 50, 51, 54–56, 58, 59, 61–64, 68–72, 74, 75, 77, 82–84, 86–90, 94]. Fifty-four studies reported minimum age (ranging from 2 to 69 years), maximum age (ranging from 7 to 87 years), and age range (2 to 87 years) [3–5, 9, 11, 16, 18, 20, 21, 30–34, 36–42, 46, 48–50, 52, 54, 55, 59, 60, 63–68, 70, 72, 74, 75, 77, 78, 80–84, 88–94]. Fifty-three studies reported mean height (ranging from 100.9 to 187.8 cm) and 52 reported height standard deviation [3, 4, 8, 9, 11, 16, 18, 22, 30–32, 35, 36, 38, 39, 41–52, 54–56, 58, 59, 61–64, 68–72, 74, 75, 84–91, 93, 94]. Fifty-four studies reported mean weight (ranging from 15.7 to 111.8 kg) and 53 reported weight standard deviation [3, 4, 8, 9, 11, 16, 18, 22, 30–33, 35, 36, 38, 39, 41–52, 54–56, 58, 59, 61–64, 68–72, 74, 75, 84–91, 93, 94]. Twenty-four studies reported BMI mean (ranging from 15.2 to 32.9 kg/m^2^), and 23 reported standard deviation [18, 20, 21, 34, 35, 41, 43–45, 47, 50, 52, 54, 55, 61, 68, 70–72, 74, 75, 89, 93, 94]. Twenty-three studies reported participant ethnicity/cultural background [3, 4, 11, 13, 31–33, 35, 40–42, 44, 55, 59–61, 63, 64, 68, 76, 77, 80, 88].

### Scanning conditions

Sixty-seven of the included studies performed 3D surface scanning of participants in bipedal stance (half bodyweight) [3, 4, 9, 11, 13, 16, 18, 20–22, 30–37, 39–45, 47, 48, 50–54, 56–59, 61–75, 78–82, 84–86, 88–95], 11 studies with partial/semi weightbearing (seated/standing) [22, 43, 52, 59, 70–72, 83, 84, 86, 90], six studies in unipedal stance (full bodyweight) [18, 57, 59, 75, 84, 90], five studies in non-weightbearing (prone/supine) [4, 30, 34, 46, 57], and two studies with external bodyweight [36, 57]. Nine studies did not report the weightbearing condition [5, 8, 38, 49, 55, 60, 76, 77, 87]. Thirty-nine studies reported performing scans bilaterally [3, 5, 9, 11, 13, 18, 20–22, 30, 31, 33, 37, 40, 43, 44, 50, 52, 53, 55, 56, 58, 59, 61, 62, 65, 69, 70, 72, 73, 76, 77, 79, 86–88, 91, 92, 95] and 32 reported unilateral scanning [4, 16, 32, 34, 35, 39, 41, 42, 45–49, 51, 63, 64, 66–68, 71, 74, 75, 78, 80–85, 89, 90, 93]. Seventeen studies provided a justification if unilateral scanning was performed (random, dominant, left, right, most painful) [16, 34, 45, 63, 64, 67, 68, 74, 75, 81, 82, 84, 85, 89, 90, 93, 94]. Thirty studies analysed the right foot only [4, 9, 16, 32, 35, 39, 41–45, 47–49, 51, 63, 64, 68–71, 78, 80–84, 89, 92, 93], 30 analysed both feet [3, 5, 8, 11, 13, 18, 21, 22, 31, 33, 37, 40, 50, 52, 53, 55, 56, 58, 59, 61, 65, 72, 73, 75–77, 86–88, 91], and three analysed the left foot only [67, 85, 90]. Forty studies reported scanning the foot in a barefoot condition [3, 9, 16, 20–22, 30, 32–34, 36, 39, 40, 43–45, 47, 48, 52–55, 59, 64–72, 74, 81, 83, 84, 86, 92, 94, 95], while one study reported the use of socks/hosiery [46]. Twenty-eight studies reported the number of scans per foot (ranging from two to 15 scans) [4, 16, 18, 21, 22, 33, 34, 36, 39, 50, 52–55, 63, 64, 66, 68, 69, 71, 72, 75, 78, 86, 88, 90–92]. Thirty-three studies reported the use of scanning markers [3, 13, 16, 32–34, 39, 40, 42, 44, 48, 50, 51, 54, 55, 59, 61, 63, 64, 67–69, 71, 72, 77, 80–84, 86, 88, 91] and 12 used markerless scanning [20, 30, 46, 47, 52, 53, 75, 79, 92–95]. Thirty studies reported the number of markers used in their scanning process, which included a range of one to 14 markers [3, 16, 32–34, 39, 40, 42, 44, 48, 50, 51, 54, 55, 59, 61, 63, 64, 67–69, 71, 72, 80–84, 86, 91]. Except for four studies [22, 34, 46, 92], static platform scanners were used. Fifty-nine studies captured at or above malleolar level [3, 8, 11, 16, 18, 22, 30–34, 37–42, 44, 46–50, 53–59, 61, 62, 64, 66–69, 72–77, 79, 81–95], six studies captured the plantar surface only [4, 20, 21, 52, 65, 78], and thirteen studies did not report the depth of scan [5, 9, 13, 35, 36, 43, 45, 51, 60, 63, 70, 71, 80].

### Scanner specification﻿s

Seventy-two of the included studies reported the 3D scanner model. Twenty-five studies used the INFOOT USB scanning system (IFU-S-01, I-Ware Laboratory Co., Ltd, Japan) [3, 8, 33, 40, 41, 44, 50, 54–56, 58, 61, 63, 64, 68, 76, 77, 80–82, 85, 87–89, 91], seven used the YETI foot scanner (Vorum Research Corporation, Canada) [38, 48, 51, 59, 66, 67, 73], four used the Microsoft® Kinect [11, 20, 21, 53], four used the 3D easy-foot-scan (OrthoBaltic, Kaunas, Lithuania) [35, 36, 47, 62], three used the FSN-2100 (Dream GP Inc., Osaka, Japan) [43, 45, 70], two used the FotoScan 3D scanner (Precision 3D Limited, United Kingdom) [89, 94], two used the FootIn3D (Elinvision, Lithuania) [18, 90], and 25 studies reported using other scanner models not used in any other study included in this review [4, 9, 13, 16, 22, 30, 34, 37, 39, 42, 46, 49, 52, 69, 71, 72, 74, 75, 78, 79, 83, 86, 92, 93, 95]. Sixty-one studies reported the scanner type. Forty-two studies stated the use of a laser scanner [3–5, 9, 18, 33, 36, 38, 40, 43–45, 47, 48, 50–52, 54–59, 61, 63–65, 67, 68, 70, 72, 73, 79–82, 84, 85, 87, 89, 91, 93], eight studies stated the use of a structured/projected light scanner [16, 34, 37, 74, 75, 78, 89, 92], 11 studies stated other scanner types (RGB-depth camera, smartphone [LiDAR], and author own custom devices) [11, 20–22, 30, 32, 39, 41, 42, 46, 86], and 18 studies did not state a scanner type [8, 13, 31, 35, 49, 53, 60, 62, 66, 69, 71, 76, 77, 83, 88, 90, 94, 95]. Forty-five studies reported scanner accuracy, with a range of < 0.2 to 3.4 mm [3–5, 9, 11, 16, 18, 21, 30, 31, 33–35, 37, 40, 44, 46, 47, 49–55, 58, 59, 62–64, 68, 69, 71, 73–75, 78, 83, 84, 86, 89, 90, 92–94]. Thirty-two studies reported scanner resolution [4, 13, 16, 18, 21, 30, 33–37, 40, 43, 46–48, 51, 56, 58, 59, 70, 72–75, 78, 82–85, 88, 90, 92]. Thirty-four studies reported scanner capture duration (ranging from 0.1 to 30 s) [3, 4, 16, 18, 21, 30, 32, 34, 37, 39, 40, 42–47, 49, 50, 52–54, 59, 63, 64, 68, 70, 72, 78, 84, 90–92, 95]. Eleven studies performed independent testing for scanner accuracy [21, 39, 49, 53, 59, 69, 71, 75, 78, 86, 91].

### Scanner reliability and calibration methods

Twenty-seven studies reported 3D scanner intra-rater (test–retest) reliability [20–22, 30–34, 37, 39, 40, 46, 49, 53, 59, 69, 71, 73, 75, 78, 82, 86, 88, 90–93]. Two studies reported scanner inter-rater reliability [90, 93]. Fourteen studies reported scanner calibration methods [22, 30, 33, 37, 39, 49, 57, 59, 78, 83, 84, 86, 89, 93].

### Foot dimensions measured

**Fig. 2:**
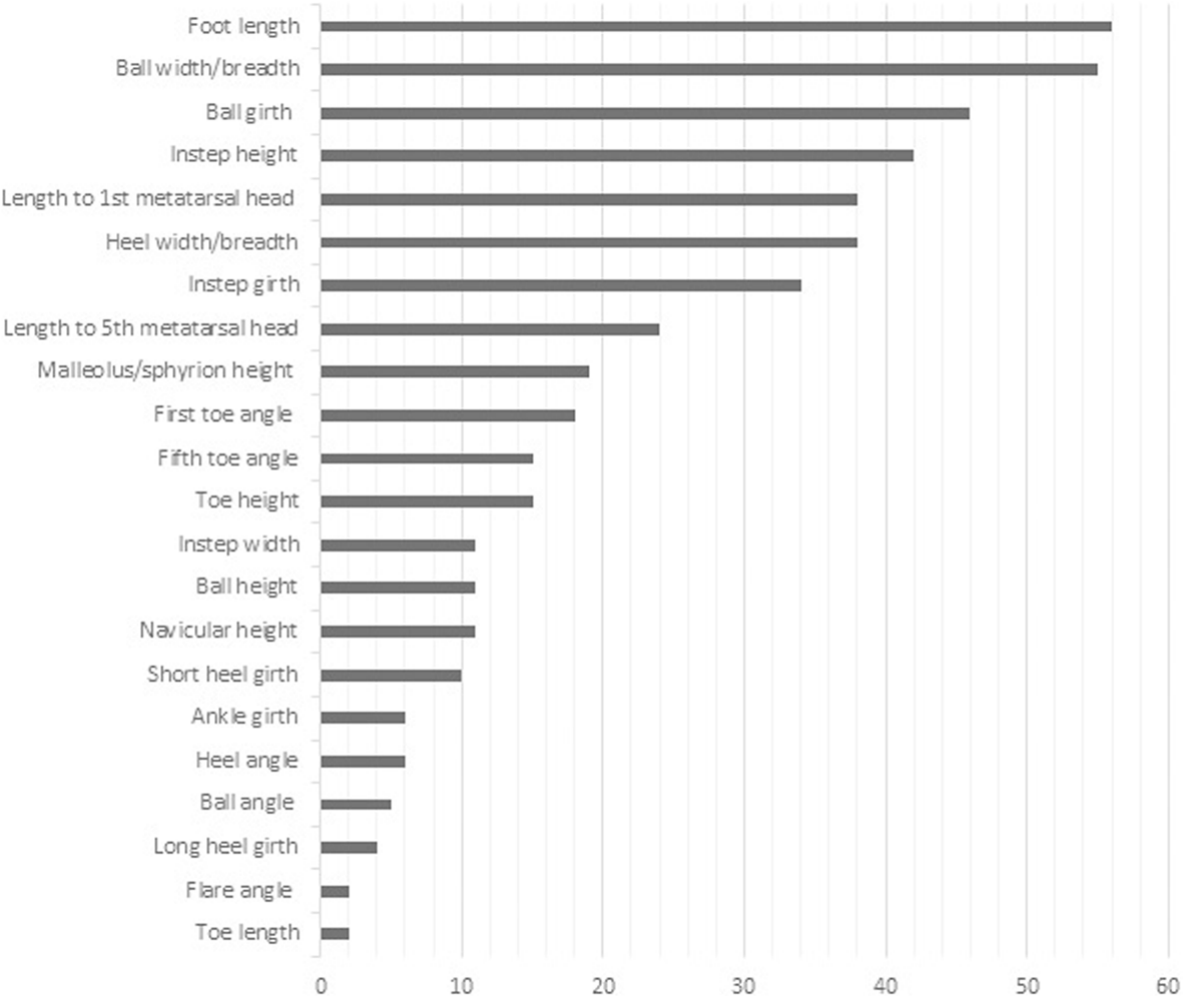
Frequency of reported 3D foot scan dimensions from the included paper﻿s

**Fig. 3:**
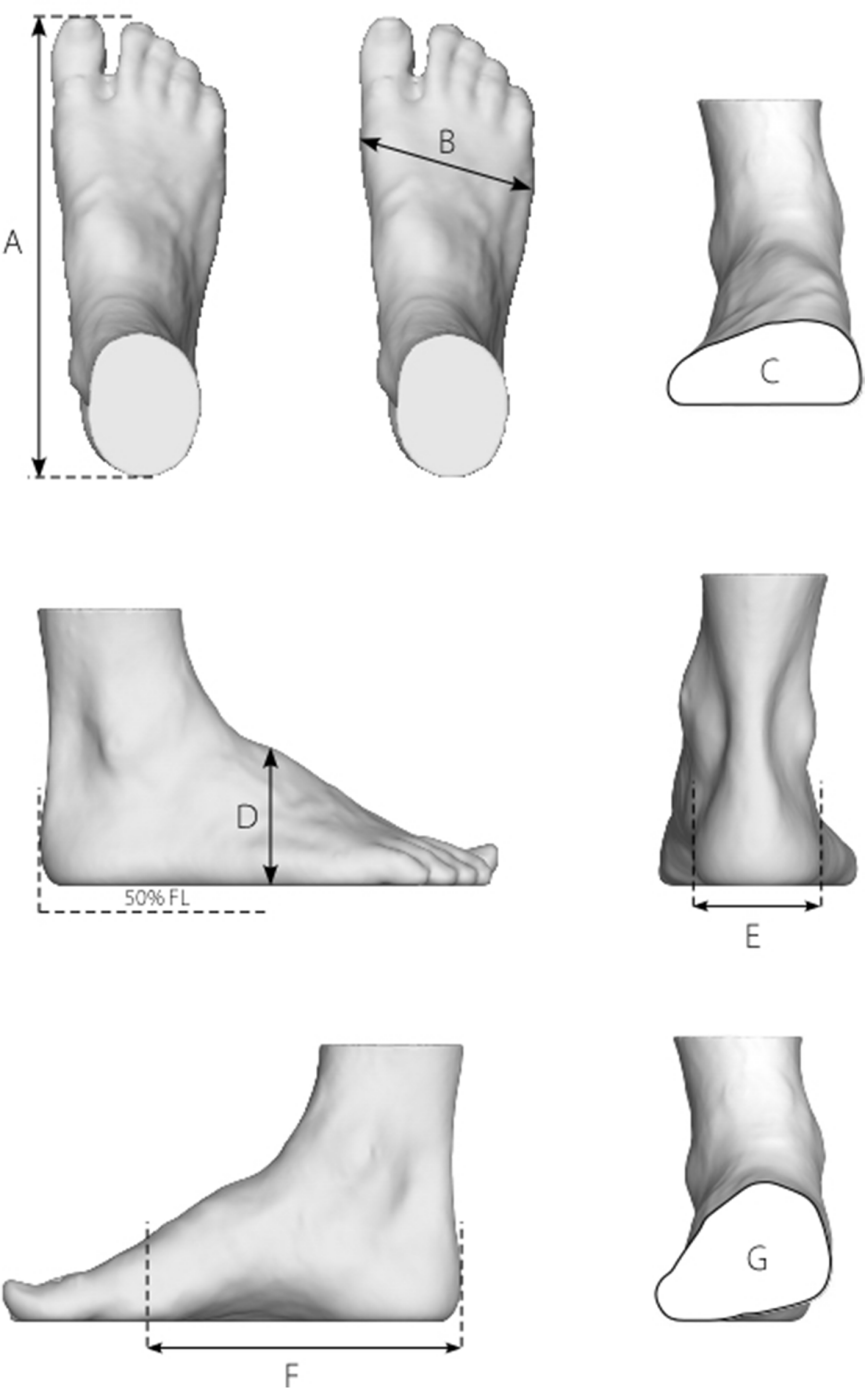
The most frequently reported 3D foot scan dimensions from the included papers. **A**: foot length (FL), **B**: ball width, **C**: ball girth, **D**: instep height, **E**: heel width, **F**: length to first metatarsal head, **G**: instep girth

A wide range of foot measures to quantify foot shape were reported within the included studies (see Fig. 2). Sixty-five studies measured foot length [3–5, 8, 9, 11, 13, 20–22, 30–38, 40–44, 46–50, 53, 55–64, 66–77, 79–82, 84, 85, 87–89, 91, 93–95], 64 studies measured ball width/breadth [3–5, 9, 11, 13, 20–22, 30–35, 37, 39–44, 46–50, 53, 55–64, 66–77, 79, 81–89, 91, 93–95], 51 studies measured ball girth [3, 5, 11, 22, 30–33, 37, 38, 40–44, 47–49, 53, 55–57, 59, 61–77, 79, 82–89, 91, 93], 51 studies measured instep height [4, 8, 13, 20, 21, 30–34, 38, 40–44, 47–50, 54, 55, 57–59, 61–64, 67–75, 78–84, 89, 91, 93–95], 43 studies measured heel width [8, 9, 13, 30–33, 40–44, 46–48, 55, 57–59, 61, 63, 64, 66–69, 71–76, 79, 81, 83–86, 89, 91, 93, 95], 43 studies measured length to first metatarsal head (medial arch length) [13, 21, 30–34, 40–44, 47, 48, 50, 54–57, 59, 61–64, 67–71, 73–76, 79, 80, 83–86, 88, 89, 91, 93], 38 studies measured instep girth [8, 22, 30–34, 37, 38, 40–44, 47–50, 55, 56, 61–64, 66–68, 72, 75–77, 79, 82, 84, 86, 87, 89, 91], 27 studies measured length to fifth metatarsal head (lateral arch length) [13, 32, 33, 40, 41, 43, 44, 47, 50, 55, 56, 61, 63, 64, 68–71, 73–75, 79, 84, 88, 89, 91, 93], 20 studies measured first toe angle [33, 35, 39–41, 43, 44, 47, 50, 61, 63, 64, 69–72, 74–76, 91], 20 studies measured malleolus/sphyrion height [8, 30–32, 41, 42, 44, 46, 48, 49, 55, 57, 59, 61, 73, 76, 81, 83, 84, 91], 19 studies measured toe height [8, 13, 32, 33, 41, 42, 57, 61, 63, 64, 68, 72, 73, 76, 81–84, 89], 17 studies measured fifth toe angle [33, 39, 40, 43, 44, 50, 61, 63, 64, 69–72, 74–76, 91], 13 studies measured instep width [21, 30, 31, 48, 56, 58, 59, 67, 78, 79, 83–85], 13 studies measured ball height [33, 37, 39, 40, 42, 50, 55, 61, 73–75, 91, 93], 13 studies measured navicular height (sitting/standing) [34, 41, 42, 50, 55, 61, 63, 69, 71, 72, 76, 80, 91], 12 studies measured short heel girth [5, 30, 32, 37, 41, 44, 48, 55, 67, 76, 82, 87], eight studies measured ankle girth [30, 32, 42, 48, 49, 73, 79, 87], six studies measured heel angle (frontal plane) [69, 71, 73, 76, 91, 94], six studies measured ball angle [32, 74, 75, 79, 83, 84], five studies measured long heel girth [30, 34, 48, 67, 86], three studies measured flare angle [11, 58, 85], and two studies measured toe length [50, 84]. Figure 3 shows the most commonly reported foot dimensions.

### IEEE and ISO reporting

Thirteen studies cited the ISO standards [33, 35, 39, 47, 49, 50, 57, 63, 64, 68, 80, 82, 88]. No studies cited the IEEE whitepaper standards.

### Processing techniques and software used

Nineteen studies reported the processing techniques used. Sixteen studies used meshing [11, 16, 18, 20, 37, 39, 49, 52, 65, 79, 82, 88, 90, 92, 94, 95], 11 studies used smoothing [20, 35–37, 42, 47, 58, 78, 85, 92, 94], six studies used scaling [18, 44, 46, 79, 82, 90], and four studies used cropping [18, 79, 85, 93].

### Statistical analysis techniques

Forty-three studies reported analysing mean differences (i.e., *t*-tests or ANOVAs) [3–5, 8, 9, 11, 22, 32–36, 39–41, 43–45, 47, 48, 50, 52, 54–56, 59–62, 68–72, 75, 76, 80, 83, 86, 87, 89, 91, 93], 24 studies reported analysing associations (i.e., Pearson’s *r*, Spearman’s *rho*, or intraclass correlation coefficients) [5, 9, 11, 16, 20, 22, 32–34, 40, 41, 43, 45, 49, 51, 52, 55, 58, 75, 78, 80, 84, 87, 91], and 20 studies reported performing cluster analysis (i.e., principal components analysis [PCA] or k-means) [11, 13, 16, 18, 20, 31, 32, 38, 42, 51, 56, 58, 63, 64, 68, 73, 78, 85, 87, 90].

## Discussion

The purpose of this scoping review was to provide a description of the methodological and statistical analysis protocols used in 3D surface scanning of the foot. Overall, the included literature highlights large variability of methods used in 3D foot scanning. We identified four key areas that showed variability and paucity of reporting between 3D scanning methods, these included the wide range of scanners used (model, type, specifications), scanner condition protocols (weightbearing condition, markers used, number of scans, unilateral/bilateral, barefoot/socks), statistical analysis techniques, and foot measures used. Additionally, the literature included a broad range of participants (height, weight, age, sex, sport, medical condition); however, studies were heavily skewed to include healthy young adults of Chinese heritage. The predominance of young adults from Chinese heritage may be due to most studies being conducted in China and using a convenience sample. Furthermore, the included studies lacked data on pathological foot shapes (hallux valgus and osteoarthritis). This underlines the need for a consistent approach in reporting methods for future 3D surface scanning research.

**Fig. 4:**
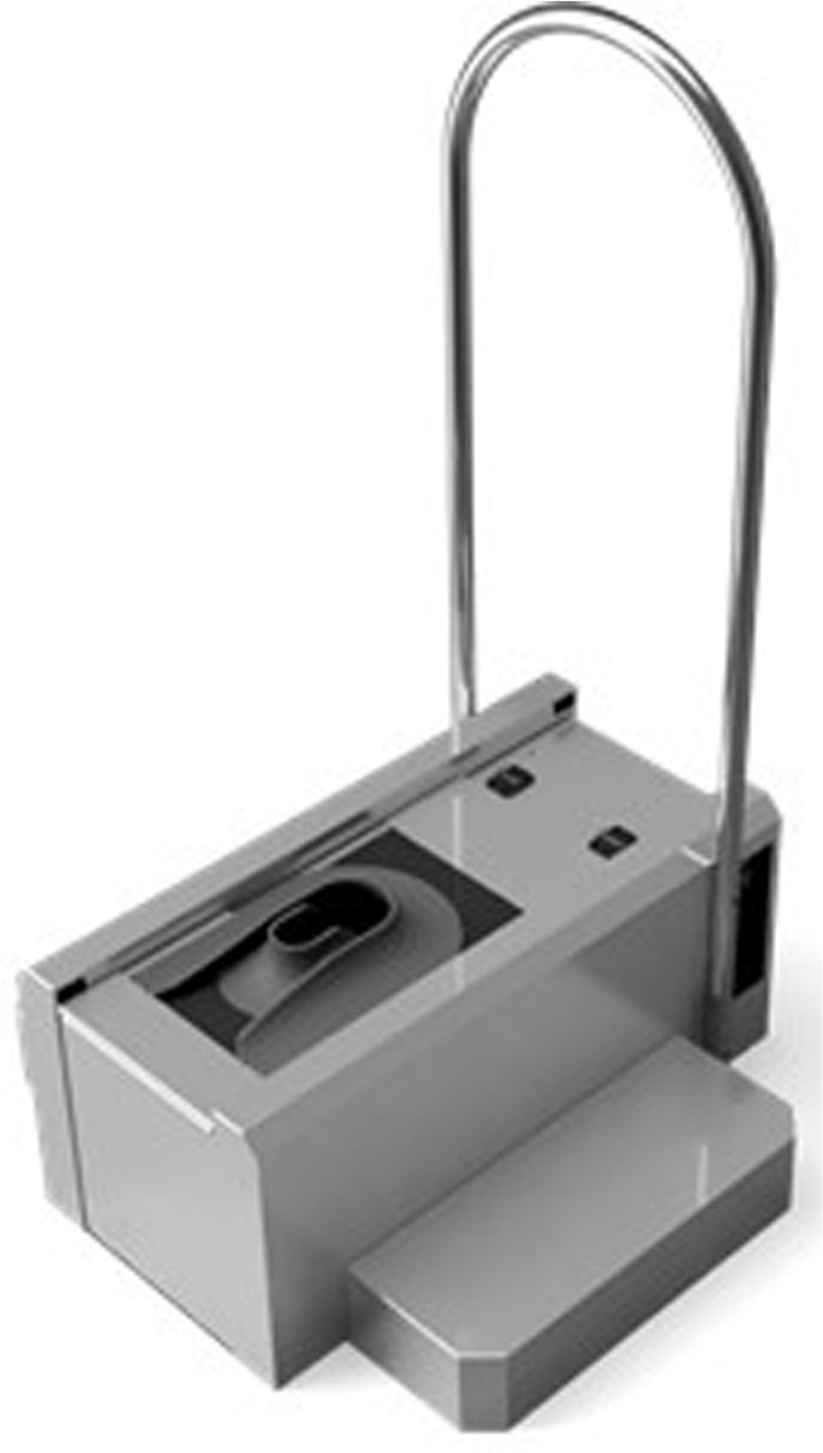
The INFOOT USB scanning system (IFU-S-01, I-Ware Laboratory Co., Ltd, Japan), the most frequently reported 3D scanner from the included papers

The available evidence incorporated several 3D scanning models and associated scanning types to collect 3D foot shape data. The most common type of scanning system was laser scanning, with the INFOOT USB scanning system (IFU-S-01, I-Ware Laboratory Co., Ltd, Japan) being the most frequently used (see Fig. 4). However, a large proportion of studies did not report the scanning type or scanner model. Authors may have not considered this detail to be of high importance to the methods or aims of the study. Additionally, another reason may have been due to the scanner being the authors’ own custom design. A likely explanation for the variability of model selection is scanner cost with several studies using low-cost scanners such as smartphone cameras [22] and the Microsoft Kinect motion sensor [53]. Across studies, 3D scanner price ranged from approximately $200USD for a depth sensor (Microsoft® Kinect) to $20,000USD for the INFOOT USB scanning system, with the latter potentially not being economically viable for many researchers. The reporting of scanner accuracy, resolution, and capture duration were also highly variable between studies. For example, scanner accuracy, resolution, and capture duration were only reported in 39 (50%), 25 (32%), and 28 (35%) studies, respectively. Additionally, resolution was reported in several different ways, these included width x height (pixels), megapixels (width x height divided by 1 million), or total point clouds at intervals per cross section of foot length (mm) [48, 56, 84]. The variability in capture duration reporting was evident between studies. Capture duration is an important specification to include in the scanner selection process as shape data distortion can be minimised with faster capture speeds [96]. Currently, it is difficult for current and future researchers to select the best suited scanner model as there is a lack of consistency and clarity in reporting of these specifications.

Scanner reliability was scarcely reported, with only 21 of the 78 (26%) studies performing test–retest reliability and 13 (17%) reporting scanner calibration methods. This may be problematic as potential technical error of measurement of the observer conducting the scan could occur, and may not be identified without reliability and scanner calibration being performed prior to data collection [88]. Similarly, there was considerable variation in the number of foot scans performed per participant. Two studies examined reliability and validity of the INFOOT 3D scanner using three repeat scans per participant [91, 97]. This showed very good to excellent reliability for most measurements and a strong correlation between the scanner and both x-ray and clinical measurements [91].

**Fig. 5:**
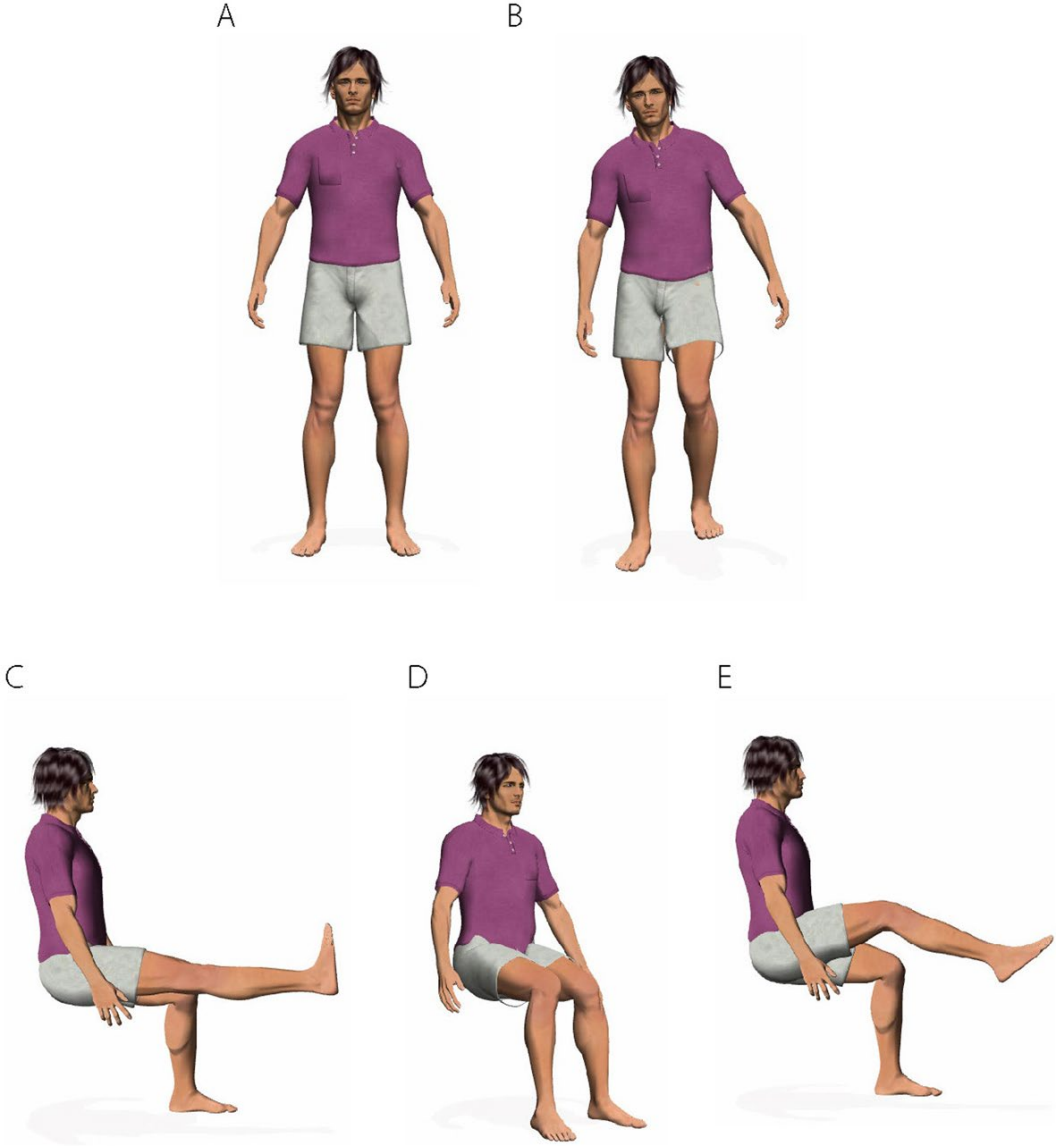
Diagrammatic representations of the most frequently reported 3D foot scanning postures from the included papers. **A**: half-weightbearing (bipedal), **B**: fully weightbearing (unipedal), **C**: non-weightbearing, **D**: partial weightbearing (seated), **E**: partial weightbearing, seated, inclined plane

Variability in weightbearing condition definitions were evident among the included studies. The description for semi/partial weightbearing and non-weightbearing were inconsistent. The IEEE define non-weightbearing as “the foot is in the air when measured not supporting any bodyweight” [24]. Several studies reported the participant in a non-weightbearing condition; however, the described position involved the participant resting their foot on the 3D scanner (in a seated or reclined position). This inconsistency would result in some weight transfer through the foot, with authors reporting this weight being up to 1% of the participant bodyweight [84]. As a result, this positioning cannot truly be classified as non-weightbearing and instead represents a partial/semi weightbearing condition. Additionally, the term ‘minimal weightbearing’ has been used by authors to describe the semi/partial weightbearing condition [90]. Since length, width, and arch height have been shown to vary in different weightbearing conditions [57] and the largest inter and intra operator foot shape differences occur in semi/partial bodyweight scans [90], it is important to have accurate descriptions of the participant scanning condition. Furthermore, there is no current definition for the external body weight condition or a specific protocol of how to collect external body weight scans. For example, authors calibrated external weight (using iron bars) and placed into vest pockets [36], while others used pressure sensors under toughened glass [57]. The IEEE definitions for weightbearing postures are shown in Fig. 5.

The types of marker placement (manual and virtual/automatic) were inconsistent between studies. Both methods have their limitations. The manual method is time consuming and relies on an experienced investigator to accurately palpate anatomical landmarks. Automatic marker placement is faster, although there may be errors during software estimation of landmark positions [75, 98]. There was also considerable variability in the number of markers used. Some studies simply used two manual markers to identify the first and fifth metatarsal heads, then used software to estimate the other landmarks (virtual and manual) [50]. Conversely, other authors used up to 14 manual markers to identify anatomical landmarks critical for shoe fitting [81]. The use of unilateral and bilateral scanning was comparable within the included studies, with 50% and 41% of studies using bilateral and unilateral scanning, respectively. Several studies found differences between the left and right foot. The differences largely involved arch height, which was theorised to be related to foot dominance [50, 70]. These findings indicate that unilateral scanning has the potential to miss key foot shape information involving the midfoot/arch area. Furthermore, this may be more apparent in populations with unilateral foot pathology (e.g., hallux and osteoarthritis).

**Fig. 6:**
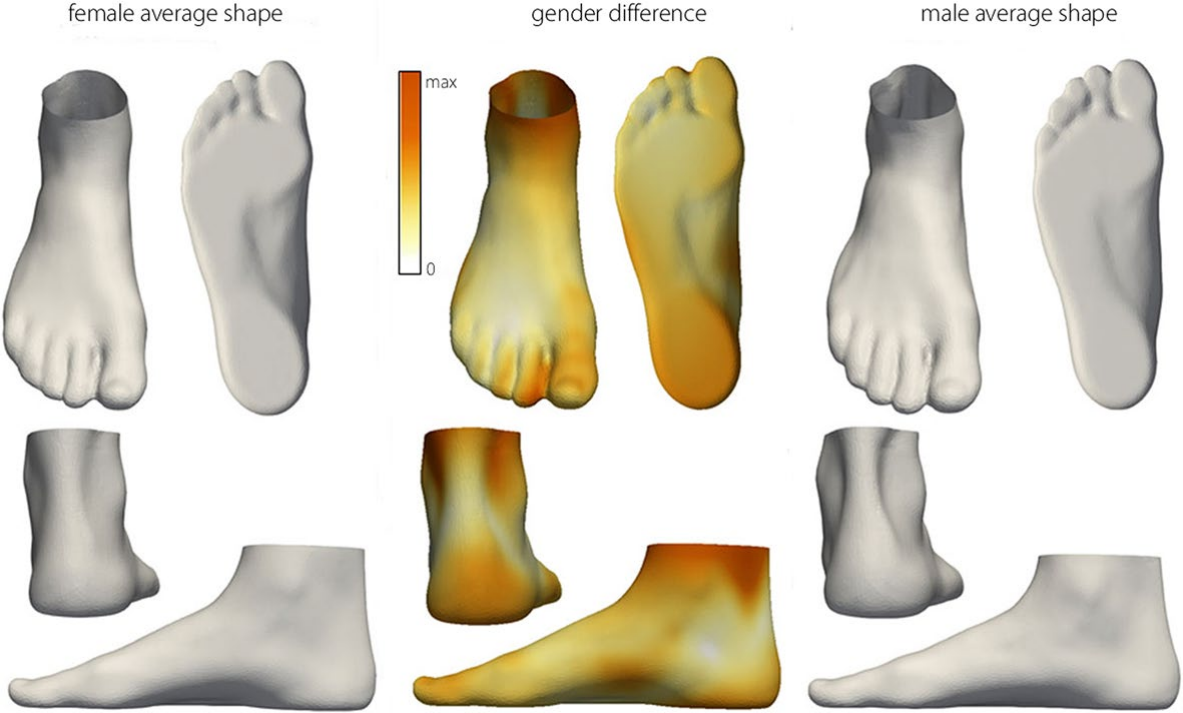
The use of principal components analysis of 3D point cloud data to identify shape differences between females and males. Image adapted from Stankovic et al. (*J Foot Ankle Res* 2018;11:8)

While all studies included in this review used 3D scanning technology and several authors applied PCA to determine foot shape, most studies performed PCA by extracting simple 1D or 2D measures (e.g., length, width, and girth measurements) from the 3D foot scan [13, 42]. This is commonly performed by predetermining the measures from all participants prior to obtaining the 3D scan, then comparing the measurement variations across the range of foot scans. This technique reduces the ability to utilise the rich shape data provided by 3D scan [99], resulting in the acquisition of incomplete foot shape data and attempting to infer 3D characteristics of the foot from 1D or 2D measurements. A more sophisticated approach was reported by Stanković et al, who collected 3D point cloud data from the scans and performed PCA by aligning mesh vertices to determine shape differences [18]. This means that the shape information that cannot be inferred from 1D or 2D measures are included to achieve a more accurate representation of the variability in foot morphology regardless of foot size (see Fig. 6).

Twenty-two scanning measures were identified that satisfied our inclusion criteria of being reported in at least two studies. There was high variability in foot measure descriptor terminology and definitions across the included studies. This resulted in multiple descriptors being used for the same foot measurement. For example, ball width (a term used by the IEEE) has been described within the included studies as ‘ball breadth’, ‘forefoot width/breadth’, ‘linear width’, ‘ball section width/breadth’, ‘metatarsophalangeal joint width’, ‘stick width’, and ‘diagonal width’, and several descriptors have been used to identify foot length from the posterior aspect of the heel to the first metatarsal head, including ‘instep length’, ‘arch length’, ‘heel-to-ball-length’, ‘ball of foot length’, ‘medial ball reach’, and ‘medial arch length’. We also found that definitions of the same foot measure varied depending on the scanner model and definition of the foot axis used. The ISO has created standards for 3D body scanning; however, foot length and width are the only included foot measures [23]. Additionally, only 13 (17%) of the included studies cited the use of these standards. The IEEE whitepaper [24] has collated several key descriptors and definitions based on various 3D scanner models to improve reporting consistency; however, no studies in this review cited the use of this resource. Furthermore, the IEEE whitepaper does not currently include a definition for ‘heel (frontal plane) angle’ and ‘navicular height’ and therefore no collective definition for these measures currently exists.

This review has several strengths. Firstly, we conducted a rigorous search strategy with the assistance of a research librarian (NP) to ensure optimum coverage of the available literature. Secondly, two independent reviewers were involved in the title and abstract screening and data extraction, with any conflicts being resolved by a third reviewer. Thirdly, we followed the Joanna Briggs Institute methodology for scoping reviews and used a standardised template for the extraction, analysis, and presentation of results [100]. However, the findings of this review should be interpreted in the context of the following key limitations. First, we did not include non-English publications in this review. Second, despite there being international standards, a wide range of foot measurement descriptors and definitions were used, so comparisons between studies were difficult. The difficulty in comparing foot descriptors and definitions was largely due to the limitations of the available literature. Third, due to limiting the included studies to ten participants, there could have been techniques missed from the excluded papers involving less than ten participants. Finally, a quality assessment of individual studies was not performed. However, this is considered optional when undertaking a scoping review and would not have been feasible considering the variability of study designs included.

### Recommendations

This review highlights the need for consistent reporting of 3D foot scanning protocols. There were inconsistencies in reporting of several key areas which were highlighted between studies, these included reporting of the equipment used (type, model, scanner specifications), scanning conditions (markers, weightbearing, number of scans, unilateral versus bilateral), and definitions and descriptions of scanner measurements. Additionally, the statistical analysis approaches used throughout the literature are highly variable with many studies utilising techniques that may not optimise the shape analysis capabilities of 3D scanning technology. Although international standards (ISO and IEEE) exist, they were not widely adopted in the included studies. Based on the findings of this review, we have developed a consistent reporting method (CRITIC 16 item checklist - **C**onsistent **R**eport**I**ng **T**hree-dimens**I**onal s**C**anning) for the identified areas above to help improve protocol transparency and reporting consistency between studies (see Table 1). The accurate reporting and improved protocol transparency has implications for holding researchers accountable for collecting reliable, repeatable, and accurate data to enhance industry products, which has never been more important in the 3D scanning space due to the increasing accessibility of 3D scanning technology.

**Table 1:** CRITIC 3D scanning checklist (**C**onsistent **R**eport**I**ng **T**hree-dimens**I**onal s**C**anning)

*No*	*Criteria*	*Item description*	☑	*Comments*
Participants				
1	Sample size	Reported or cited in manuscript	❐	
2	Men/women	Reported or cited in manuscript	❐	
Equipment				
3	Scanner model	Scanner name/model	❐	
4	Scanner type	Laser, structured light etc	❐	
5	Scanner resolution		❐	
6	Scanner accuracy		❐	
7	Scanner capture duration (secs)		❐	
8	Scanner calibration methods	Reported or cited in manuscript	❐	
9	Scanner reliability	Reported or cited in manuscript	❐	
10	Markers used	Number, placement—automated or manual or both	❐	
11	Number of scans	Number of scans reported per foot	❐	
Participant condition				
12	Weightbearing	According to IEEE standards – bipedal, unipedal, partial/semi weightbearing, non-weightbearing, external bodyweight	❐	
13	Barefoot/socks		❐	
14	Unilateral/bilateral scanning	Justification provided if unilateral scanning performed	❐	
Measurements				
15	Foot measures used	Definitions according to IEEE standards	❐	
Post processing				
16	Software used	Type, Automated/manual/both – please specify in comments	❐	

## Conclusions

The paper provides an overview of 3D foot scanning methodological and statistical analysis protocols. It can be concluded that inadequate reporting and lack of consistency in 3D foot scanning methodological and statistical analysis protocols exists. Despite the availability of international standards for 3D foot scanning, few studies utilised this resource and consequently there is a lack of homogeneity between scanning protocols. Additionally, the need for utilisation of optimal statistical analysis techniques of a diverse population group to quantify foot shape is needed. The improved consistency of 3D foot scanning methodologies and utilisation of more sophisticated statistical analysis techniques could enhance data pooling and collaboration between researchers. As a result, larger sample sizes, diversification of population groups, and simultaneous data collection could be obtained to enhance the quantification of foot shape and facilitate the development of improved orthotic and footwear products.

## Data Availability

Data sharing not applicable to this article as no data-sets were generated or analysed during the current study.
